# Stereotactic radiotherapy as a valuable therapeutic procedure for controlling aldosterone-secreting adrenocortical carcinoma

**DOI:** 10.20945/2359-4292-2023-0441

**Published:** 2024-07-01

**Authors:** Livia Mara Mermejo, Renato Heron Zanella, Larissa Cocicov, Carlos A. Fernandes Molina, Silvio Tucci, Jorge Elias, Valdair Francisco Muglia, Gustavo Arruda Viani, Paula C L Elias, Ayrton Custodio Moreira, Margaret de Castro

**Affiliations:** 1 Departamento de Clínica Médica Faculdade de Medicina de Ribeirão Preto Universidade de São Paulo São Paulo SP Brasil Departamento de Clínica Médica, Faculdade de Medicina de Ribeirão Preto, Universidade de São Paulo, São Paulo, SP, Brasil; 2 Departamento de Cirurgia e Anatomia Faculdade de Medicina de Ribeirão Preto Universidade de São Paulo São Paulo SP Brasil Departamento de Cirurgia e Anatomia, Faculdade de Medicina de Ribeirão Preto, Universidade de São Paulo, São Paulo, SP, Brasil; 3 Departamento de Radiologia, Hematologia e Oncologia Faculdade de Medicina de Ribeirão Preto Universidade de São Paulo São Paulo SP Brasil Departamento de Radiologia, Hematologia e Oncologia, Faculdade de Medicina de Ribeirão Preto, Universidade de São Paulo, São Paulo, SP, Brasil

## Abstract

Aldosterone-secreting adrenocortical carcinomas (ACCs) are rare and usually present as large tumors. The only potentially curative treatment for ACC is surgical resection. However, surgery may be unfeasible in some patients who have multiple comorbidities or decline the procedure. We describe herein the case of a 44-year-old man with aldosterone-secreting ACC who declined surgery because of religious convictions. As a Jehovah’s Witness, the patient was concerned about requiring blood transfusion during surgery. Treatment with mitotane was started but interrupted due to hepatotoxicity. Subsequently, the patient was successfully treated with stereotactic ablative radiotherapy (SABR). After SABR, the patient had progressive improvement of pain, reduction in antihypertensive drugs, control of blood pressure and hypokalemia, normalization of serum aldosterone and renin levels, and reduction in tumor size and weight. To our knowledge, this is the first report of a patient with a pure aldosterone-secreting ACC who received SABR. The patient’s response was substantial, showing that SABR could be considered as an alternative local treatment for aldosterone-secreting ACCs when surgery is unfeasible.

## INTRODUCTION

Adrenocortical carcinomas (ACCs) are a rare malignancy with an annual incidence within the population of 1-2 cases per million individuals (1-3). This type of tumor follows a heterogeneous clinical course, with outcomes varying widely, but typically showing a poor prognosis (1). Approximately 60% of all ACCs are hormonally active; the remaining 40% are identified incidentally or after complaints related to tumor growth and mass effects, such as metastatic disease or abdominal or back pain (1,2). Approximately 40% of patients with symptomatic ACC have Cushing’s syndrome, 24% present both Cushing’s syndrome and virilization concurrently, and only 2.5% have pure aldosterone-secreting ACCs (4). The first case of aldosterone-secreting ACC was documented in 1955, involving a man with hypertension who developed increasing weakness due to hypokalemia (5). These symptoms are the most common manifestations of primary aldosteronism due to adrenocortical adenoma or carcinoma, whose clinical presentation may often be challenging to differentiate (4). The management of ACC involves surgical resection for localized disease, with adjuvant mitotane as a therapeutic option (1,6,7). However, some patients are unable to undergo surgery because of multiple comorbidities or personal decisions. For advanced or metastatic ACC that cannot be completely resected, treatment options are limited and may include mitotane alone or combined with chemotherapy. Notably, these treatments have associated toxicities and may offer limited survival benefits (1,6,7).

We describe herein a patient with aldosterone-secreting ACC who declined surgery due to religious convictions but was successfully treated with stereotactic ablative radiotherapy (SABR).

## CASE REPORT

A 44-year-old man was referred to the University Hospital at Ribeirão Preto Medical School with complaints of back pain, muscular weakness, and hypokalemia. A year prior, he had received a diagnosis of hypertension that proved to be resistant to antihypertensive drugs. The patient presented progressive weight loss (10 kg) and reduced strength in lower limb muscles. He had a body mass index of 25 kg/m^2^, blood pressure of 180/120 mmHg, and heart rate of 72 bpm. No signs of cortisol or androgen excess, peripheral edema, or palpable abdominal mass were observed. Blood tests showed levels of sodium of 143 mmol/L (normal range [NR] 135-145 mmol/L), potassium of 1.2 mmol/L (NR 3.5-5 mmol/L), creatine phosphokinase (CPK) of 9,928 U/L (NR 24-195 U/L), elevated hepatic enzymes (aspartate aminotransferase [AST] 10 times above the upper limit of normal [ULN] and alanine aminotransferase [ALT] 2.6 times above the ULN), and metabolic alkalosis.

**Figure 1:**
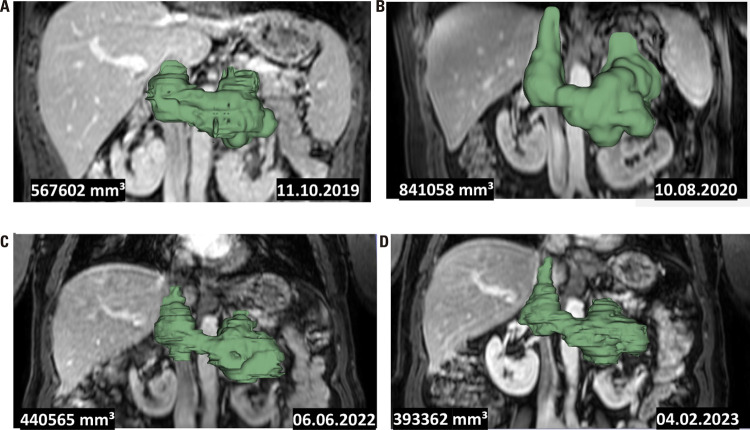
Abdominal computed tomography images analyzed using the software 3D Slicer, Version 5.0.2 (https://www.slicer.org/) to depict the tumor volume regression over time. The images depict the tumor volume (in mm3) at the time of diagnosis (A), immediately before stereotactic ablative radiotherapy (SABR) (B), and 18 months (C) and 27 months (D) after SABR treatment.

The patient developed rhabdomyolysis in the setting of profound hypokalemia as a result of renal tubular acidosis induced by an antiinflammatory medication. Due to abdominal pain and elevated liver enzymes, an abdominal computed tomography (CT) scan was performed, revealing a heterogeneous 10 x 8 x 6 cm left adrenal mass compressing the left renal vein, invading the inferior vena cava and extending to the caudate lobe of the liver. The tumor volume was 567 cm^3^ as analyzed by the software 3D Slicer, Version 5.0.2 (https://www.slicer.org/) (8) (Figure 1A). A chest CT scan and bone scintigraphy were normal. Basal serum aldosterone levels were 75 ng/dL and 72 ng/dL (NR 3-30 ng/dL), and renin levels were < 2 mIU/L and 6 mIU/L (NR 5-45 mIU/L). Levels of urinary metanephrine and normetanephrine were within normal limits (93 µg/24h [NR 26-230 µg/24h] and 408 µg/24h [NR 44-450 µg/24h], respectively). Plasma cortisol levels before and after 1 mg of dexamethasone were 9.5 µg/dL (NR 5-20 µg/dL) and 2 µg/dL (NR <1.8 µg/dL), respectively. Morning plasma adrenocorticotropic hormone (ACTH) level was 16 pg/mL (NR 10-50 pg/mL). Androstenedione, testosterone, dehydroepiandrosterone sulfate (DHEA-S), and 17-hydroxyprogesterone (17-OHP) levels were within the reference range. The patient’s family history was negative for hypertension and adrenal diseases.

After biochemical evaluation confirmed primary aldosteronism, the patient was started on spironolactone, with the dose gradually escalated to 400 mg/day, alongside potassium supplementation. Subsequently, there was marked clinical improvement, accompanied by normalization of CPK levels and liver enzymes. The patient was also taking amlodipine 10 mg/day and hydralazine 300 mg/day.

A probable diagnosis of aldosterone-secreting ACC was considered, prompting the recommendation for open adrenalectomy. However, the patient, identifying as a Jehovah’s Witness, declined the proposed surgery due to concerns over the potential need for a blood transfusion. He was then started on treatment with mitotane 1 g/day but developed rapidly increasing liver enzyme levels (serum ALT twice above the ULN and gamma-glutamyl transferase 9.7 times above the ULN), and the treatment was interrupted after 2 months. A regimen adding etoposide, doxorubicin, and cisplatin (EDP) to mitotane was not considered due to hepatic toxicity and the risk of myelosuppression, which would require blood transfusion.

A control abdominal magnetic resonance imaging (MRI) obtained 10 months later showed that the tumor mass had increased (12.3 x 8 x 9 cm, volume 841 cm^3^) and extended to the left kidney (Figure 1B). Since the patient had declined surgery, we recommended a core biopsy of the tumor to confirm the histological diagnosis and formulate a treatment plan. The patient agreed to undergo a CT-guided percutaneous core biopsy, which confirmed the diagnosis of aldosterone-secreting ACC. The biopsy report indicated the presence of three criteria of the Weiss score (greater than one-third diffuse architecture, less than 25% of clear cell component, and microscopic necrosis). However, evaluation of three other criteria (venous invasion, capsular infiltration, and sinusoidal invasion) was unfeasible due to the specimen’s small size. Immunohistochemistry revealed positivity for vimentin, inhibin, and MART-1/Melan A. A molecular investigation for the *TP53* p.R337H variant was negative (3).

The patient underwent SABR, receiving a total dose of 35 Gy in five daily fractions delivered in three dose levels: 25 Gy to the adrenal mass, vena cava, and left kidney vein/artery (because of the invasion), 30 Gy restricted to the adrenal mass, and 35 Gy delivered to a central region of interest in the adrenocortical mass. After SABR, the patient’s pain improved progressively, and he achieved control of his blood pressure levels, requiring a lower dose of antihypertensive medications (based on a defined daily dose decrease from 10.3 to 7.5). He also presented significant (p<0.0001) improvement in serum potassium levels, which increased from a median of 1.8 mmol/L (interquartile range [IQR] 1.3-1.9 mmol/L) before diagnosis and a median of 3.6 mmol/L (IQR 3.2-3.7 mmol/L) before radiotherapy to a median of 4.2 mmol/L after radiotherapy (IQR 3.8-4.7 mmol/L). Of note, the patient was on spironolactone during measurements of potassium levels, except for the measurement done before diagnosis. After SABR treatment, the patient returned to work and decreased emergency visits due to pain and hypokalemia.

**Figure 2:**
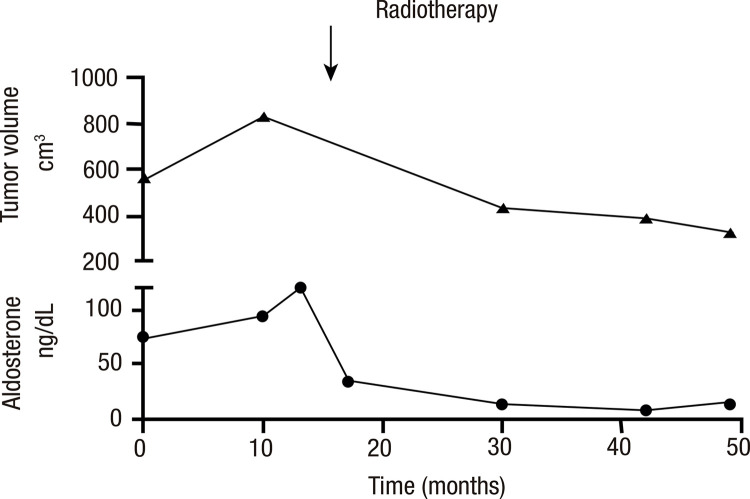
Line graph illustrating the progression of tumor volume (in cm3) and aldosterone levels (in ng/dL) over time, from diagnosis through posttreatment with stereotactic ablative radiotherapy. The graphs are presented concurrently to compare their respective trajectories.

Follow-up abdominal MRIs obtained 18 months and 27 months after SABR showed a reduced tumor volume (440 cm^3^ and 393 cm^3^, respectively) (Figures 1C and 1D). After SABR, the tumor mass reduced proportionally to the biochemical improvement, as shown by the decrease in serum aldosterone levels parallel to the increase in renin levels (Figure 2). During the preparation of this report, the patient had an overall survival of 1,490 days. During evaluation in the prior month, he no longer required potassium replacement and was taking amlodipine 10 mg/day, hydralazine 100 mg/day, and spironolactone 200 mg/day. Serum levels of basal aldosterone were 15.1 ng/dL and 15.2 ng/dL (NR 3-30 ng/dL), renin 22.4 mIU/L and 9.3 mIU/L (5-45 mIU/L), and potassium 4.6 mmol/L and 3.8 mmol/L (3.5-5 mmol/L). Although the use of spironolactone may interfere with the measurements of aldosterone and renin, the patient had taken this drug since diagnosis. At 35 months after SABR treatment, the tumor volume was stable at 330 cm^3^(Table 1).

**Table 1:** Parallel longitudinal trends in tumor mass reduction and biochemical improvement observed before and after stereotactic ablative radiotherapy in a patient with aldosterone-secreting adrenocortical carcinoma

	At diagnosis	10 months after diagnosis	3 months after SABR	8 months after SABR	18 months after SABR	27 months after SABR	35 months after SABR
Aldosterone (ng/dL)	75 and 72	93	119	35	13	8	15
Renin (mIU/L)	<2 and 6	7.8	15	25	25	11	10
Spironolactone dose (mg/day)	None	400	400	400	400	300	200
Tumor volume (cm^3^)	567	841	-	-	440	393	330

Abbreviation: SABR, stereotactic ablative radiotherapy.

## DISCUSSION

To the best of our knowledge, this is the first case report detailing a longitudinal effective use of SABR for the treatment of aldosterone-secreting ACC in a patient who declined surgery due to religious convictions.

Isolated aldosterone-secreting ACCs are exceedingly rare. This rarity accounts for the limited information on the biological behavior of these tumors, as well as the treatment options and patient survival rates. Seccia and cols. published a review of 60 patients with aldosterone-producing ACCs (4). The authors found the patients had a median age of 44 years (range 17-79 years), and the tumors had a median diameter of 7 cm (range 2.5-15.0 cm) and a median weight of 248 g (range 6.3-1,250 g). More than half of the patients had increased aldosterone levels (14 times above the NR) and suppressed renin levels (4). All but three patients had hypertension and hypokalemia, with the latter condition being linked to symptoms of weakness and diffuse muscular pain. Other clinical features included pyrexia and weight loss associated or not with symptoms of virilization or Cushing’s syndrome (1). Our patient had a pure aldosterone-producing ACC, and his clinical characteristics were aligned with those presented in the review by Seccia and cols. Our patient was 44 years old at diagnosis and had hypertension, diffuse muscular weakness, marked hypokalemia, very high aldosterone levels, and suppressed renin levels. His tumor measured 10 cm in diameter; he had no metastasis at diagnosis and reported persistent back pain, which is one of the most common symptoms associated with large aldosterone-secreting ACCs (1).

Regardless of the ACC subtype, the most important prognostic factors associated with these tumors are stage at presentation and completeness of surgical resection (1,9,10). However, surgery has not been a definitive treatment in most patients, as occult micrometastases may be present even at the early stages of the disease (1,9). Indeed, Seccia and cols. reported metastases in 10% of all aldosterone-producing ACCs at diagnosis and in an additional 48% of the cases during follow-up (4). Kendrick and cols. have shown an increased risk of perioperative death in patients with aldosterone-secreting ACCs (11). In their study at the Mayo Clinic, aldosterone-secreting ACCs comprised only 11% of all ACC types over 44 years but accounted for 33% of all perioperative deaths related to these tumors (11).

Our patient, a Jehovah’s Witness, declined surgery due to a concern that it involved a high risk of requiring a blood transfusion. One alternative to surgical treatment was mitotane, administered as neoadjuvant therapy. No clear results were observed, but mitotane had to be discontinued after a short period because of increasing levels of liver enzymes. Conventional radiotherapy would be an alternative option in the present case. Although conventional radiotherapy is not a primary treatment for ACC, it may be used in the adjuvant setting to manage surgically resected and nonsecreting ACCs locally. Given the therapeutic dilemmas presented by this case, we decided to proceed with treatment using SABR, which our patient accepted. By delivering high ablative doses per fraction with accurate precision while sparing surrounding normal tissues and organs, SABR has several advantages over conventional radiotherapy. The decision for SABR was based on the effectiveness of adjuvant radiotherapy in reducing the risk of local recurrence and the excellent local control (about 80% in 1 year) in treating adrenal metastases from several other primary tumors (12,13).

Data on adjuvant radiotherapy for ACCs are conflicting, even in those associated with Cushing’s or virilization syndromes. One study examining radiotherapeutic effects on ACC outcomes found no improvement in overall or disease-specific survival (14). One disadvantage of radiotherapy is that it hinders the detailed pathological characterization of the tumor. However, some benefits of radiotherapy for ACC have been reported. A meta-analysis with 132 patients with ACC comparing surgery with *versus* without adjuvant radiotherapy found that adjuvant radiotherapy compared with surgery alone was better for local control and reduced the relative risk of relapse (13). A recent retrospective study analyzing 80 patients who had received radiotherapy for advanced ACC in five European centers found that radiotherapy was effective when applied in adequate dosages. The authors concluded that radiotherapy should be considered more frequently as a treatment option in advanced disease and not only as a palliative alternative (15). The study had no information on patients with aldosterone-secreting ACC.

Our patient was treated with SABR and, at the time of writing of this case report, had reached 4 years of survival since the diagnosis with optimal disease control and decreased tumor volume (841 cm^3^ before SABR and 393 cm^3^and 330 cm^3^ after SABR). Of note, the median overall survival of patients with aldosterone-secreting ACCs is only 546 days (95% confidence interval [CI] 240-851 days), and the median time to recurrence or death is 212 days (95% CI 29-395 days) (4). Recently, Zhang and cols. updated the aldosterone-producing ACCs database created by Seccia and cols., reporting a median survival time of 1,460 days and a median time for tumor recurrence or death of 365 days (16). The observed increase in survival rate probably resulted from earlier diagnosis and more advanced treatment of this disease with the addition of more recent cases to the database (16).

The occurrence of necroptosis induced by a high dose of radiation delivered over a relatively short time (five fractions) can explain the long-term local control achieved with SABR. This process increases vascular permeability, aggravates platelet aggregation and thrombosis formation, and induces blood vessel injury and ischemia, leading to tumor necrosis. The tumor repair mechanism cannot mend the massive DNA damage caused by radiation, resulting in cell death. Additionally, SABR directly or indirectly activates inflammatory cytokines and recruits immune cells, resulting in an intense CD8(+) T-cell tumor infiltration, leading to immunogenic cell death (17). We hypothesized that these factors altogether may have been responsible for the long-term local control with SABR in lieu of surgery in our patient’s large aldosterone-secreting ACC. When feasible, SABR may be considered a preferred treatment option because it involves a shorter overall treatment period.

In conclusion, this report highlights the use of local SABR radiotherapy in a case in which surgery – the main therapeutic option for ACC – is unfeasible. The findings reported herein unveil a new therapeutic possibility for conditions with a poor prognosis and limited treatment options like unresectable ACCs.

Patient consent and ethical committee approval: the patient signed a written informed consent agreeing with the publication of this case report. The study was approved by the Ethical Committee of the Ribeirão Preto Medical School, University of São Paulo (protocol number: 68380422.3.0000.5440).
